# Identification and Analysis of Blood Gene Expression Signature for Osteoarthritis With Advanced Feature Selection Methods

**DOI:** 10.3389/fgene.2018.00246

**Published:** 2018-08-30

**Authors:** Jing Li, Chun-Na Lan, Ying Kong, Song-Shan Feng, Tao Huang

**Affiliations:** ^1^Department of Rehabilitation, The Second Xiangya Hospital, Central South University, Changsha, China; ^2^Department of Neurosurgery, Xiangya Hospital, Central South University, Changsha, China; ^3^Institute of Health Sciences, Shanghai Institutes for Biological Sciences, Chinese Academy of Sciences, Shanghai, China

**Keywords:** osteoarthritis, blood, gene expression, signature, support vector machine, minimal redundancy maximal relevance, incremental feature selection

## Abstract

Osteoarthritis (OA) is a complex disease that affects articular joints and may cause disability. The incidence of OA is extremely high. Most elderly people have the symptoms of osteoarthritis. The physiotherapy of OA is time consuming, and the chances of full recovery from OA are very minimal. The most effective way of fighting OA is early diagnosis and early intervention. Liquid biopsy has become a popular noninvasive test. To find the blood gene expression signature for OA, we reanalyzed the publicly available blood gene expression profiles of 106 patients with OA and 33 control samples using an automatic computational pipeline based on advanced feature selection methods. Finally, a compact 23-gene set was identified. On the basis of these 23 genes, we constructed a Support Vector Machine (SVM) classifier and evaluated it with leave-one-out cross-validation. Its sensitivity (Sn), specificity (Sp), accuracy (ACC), and Mathew's correlation coefficient (MCC) were 0.991, 0.909, 0.971, and 0.920, respectively. Obviously, the performance needed to be validated in an independent large dataset, but the in-depth biological analysis of the 23 biomarkers showed great promise and suggested that mRNA surveillance pathway and multicellular organism growth played important roles in OA. Our results shed light on OA diagnosis through liquid biopsy.

## Introduction

Osteoarthritis (OA) is a complex disease that affects articular joints and may cause disability (Appleton, 2017). In the USA, 14 million people have symptomatic knee osteoarthritis (KOA) (Vina and Kwoh, 2017). Approximately 10–20% adult have OA (Bay-Jensen et al., 2018). Although OA is considered a disease primarily for the elderly, nowadays, more than half of patients with OA are under 65 years old. More and more young people show the symptoms of OA. The physiotherapy of OA is time consuming, and the chances of full recovery from OA are very minimal (Nelson, 2017). The most effective way of fighting OA is early diagnosis and early intervention. However, usually at early stage when OA is treatable, the patients often ignore the symptoms and are reluctant to go to the doctor for consultation (Nelson, 2017). When OA becomes serious, it is too difficult to treat this illness.

Blood is a vehicle for mRNAs from different tissues (Budd et al., 2017). It has been widely used for the early detection of various cancers (Zhang et al., 2017) and predictions of drug responses (Huang et al., 2008; Zhang et al., 2012). As a complex disease, the occurrence and development of OA involves changes to the mRNA (Steinberg et al., 2017). The blood flow under the subchondral bone (Aaron et al., 2018) may carry the signal of OA (Fotouhi et al., 2018). It can be detected when the mRNA level changes in blood (Budd et al., 2017). If so, then the detection of OA will be much easier and more accurate. In fact, there have been several studies of blood biomarkers for OA (Ramos et al., 2014; Feng et al., 2015; Ahmed et al., 2016; Bay-Jensen et al., 2018; Costa-Cavalcanti et al., 2018). For example, Ramos et al. demonstrated that the mRNA expression of apoptotic pathways was significantly different in the blood of patients with OA (Ramos et al., 2014). Bay-Jensen et al. reported the use of biochemical markers for OA, which measured the turnover of joint tissue or the inflammatory status (Bay-Jensen et al., 2018).

To quantify the cartilage turnover, several discovered biomarkers were used, such as PIIANP, CTX-II, ARGS, COMP, and C2C. In serum, PIIANP and CTX-II were found to be associated with OA progression by Osteoarthritis Initiative (OAI) Study of FNIH (Foundation for the National Institutes of Health; Kraus et al., 2017). ARGS was found to be associated with pain in anterior cruciate ligament injury patients (Wasilko et al., 2016). COMP was highly expressed in synovial fluid of patients with OA (Lorenzo et al., 2017). C2C was significantly different among patients with OA with no sign of cartilage damage, early signs of OA, and radiographic OA, and it was highly expressed in the patients with radiographic OA (Schaefer et al., 2017). In addition, there were biomarkers for synovial inflammation and fibrosis, such as C1M, C3M, and CRPM. They were positively correlated with elderly symptomatic OA (Martel-Pelletier et al., 2016).

Unfortunately, many of these biomarkers were for synovial fluid and most of them were only differentially expressed. Such qualitative biomarkers cannot be used in clinical settings directly, and for this reason, a blood biomarker-based quantitative classifier was the ideal model.

To build such a useful model, we reanalyzed a publicly available dataset from Ramos et al. (2014), which included the blood gene expression profiles of 106 patients with OA and 33 control samples with advanced feature selection methods, such as minimal redundancy maximal relevance (mRMR) and incremental feature selection (IFS), instead of a conventional statistical test. We identified 23 blood gene expression biomarkers. On the basis of these 23 genes, we constructed a Support Vector Machine (SVM) classifier and evaluated its performance with Leave-One-Out Cross Validation (LOOCV). The sensitivity (Sn), specificity (Sp), accuracy (ACC), and Mathew's correlation coefficient (MCC) were 0.991, 0.909, 0.971, and 0.920, respectively. In addition, we performed in-depth biological analysis of the 23 biomarkers. They were involved in the mRNA surveillance pathway and multicellular organism growth. Not only was a quantitative classifier constructed, but also the underlying mechanisms of OA occurrence and progression were revealed.

## Materials and methods

### The blood gene expression profiles of osteoarthritis and control samples

We downloaded the blood gene expression profiles of 106 OA and 33 control samples from the Gene Expression Omnibus (GEO) database under the accession number of GSE48556 (Ramos et al., 2014). The gene expression levels were measured using Illumina HumanHT-12 V3.0 expression beadchip. There were 48,802 probes corresponding to 25,159 genes. The probes representing the same gene were averaged, and the gene expression profiles of OA and control samples were quantile-normalized.

Unlike Ramos's study (Ramos et al., 2014), which identified 694 genes with adjusted *p*-value smaller than 0.05 using linear regression analysis and then narrowed down the genes to a short list using functional annotation, we aimed to develop an automatic analysis pipeline that minimized human intervention and avoided the hand-picking during biomarker selection. Despite the great performance achieved by Ramos et al. (2014), we believe that there are other actionable biomarkers which may function in a different way and we are trying to find them with advanced feature selection methods.

### Mutual information-based feature ranking

Identifying the phenotype-associated features is one of the basic problems in bioinformatics, and for different problems, there are different solutions (Huang et al., 2008; Cai et al., 2010; Zhang et al., 2012, 2015, 2016, 2017; Li et al., 2014; Chen et al., 2018a; Wang et al., 2018). For identifying differentially expressed genes (DEG), the most widely used methods are the *t-*test, significance analysis of microarrays (SAM; Tusher et al., 2001), and linear regression as performed by Ramos et al. (2014). However, usually such statistics-based methods will identify too many DEG than we require. The redundancy between DEG is extremely high. Many genes have very similar expression patterns.

Unlike DEG, we needed a smaller number of signature genes that can be applied in clinical settings. Therefore, we adopted a mutual information-based method, i.e., mRMR (Peng et al., 2005), which has been widely used in feature ranking (Niu et al., 2013; Zhao et al., 2013; Zhou et al., 2015; Zhang et al., 2016; Li and Huang, 2017; Liu et al., 2017). It considers both the relevance between features and sample labels and the redundancy among features and has been proven to be an effective feature selection method, especially for gene expression analysis (Qin et al., 2012; Zhang et al., 2014b, 2017, 2018; Zhang Y. et al., 2014; Li et al., 2015; Zhou et al., 2015; Wang et al., 2016; Song et al., 2017; Chen et al., 2018b). The method works like this: let us use Ω to denote all the 25,159 genes, Ω_*s*_ to denote the selected gene set that includes m genes, and Ω_*g*_ to denote the n genes that will be evaluated, and one of them will be selected.

First, the relevance of gene *g* from Ω_*g*_ with sample labels *l* was measured using mutual information (*I*) (Sun et al., 2012; Huang and Cai, 2013):

(1) $$\begin{array}{r} I\left(g,l\right) \end{array}$$

As the mutual information can only be calculated between categorical variables, the expression levels of each gene were discretized with the thresholds of mean minus standard deviation and mean plus standard deviation.

Then, the redundancy of gene *g* with selected gene set Ω_*s*_ was quantified:

(2) $$\begin{array}{r} \frac{1}{m}\left(\sum_{g_{i}\in \Omega_{s}}I\left(g,g_{i}\right)\right) \end{array}$$

As we wanted to maximize the relevance and minimize the redundancy, the optimization goal can be characterized as follows and the best gene form Ω_*g*_ will be selected:

(3) $$\begin{array}{r} {\text{max}}_{g_{j}\in \Omega_{g}}\left[I\left(g_{j},l\right)-\frac{1}{m}\left(\sum_{g_{i}\in \Omega_{s}}I\left(g_{j},g_{i}\right)\right)\;\right]\;\left(j=1,2,\ldots ,n\right) \end{array}$$

After n rounds of optimization, a ranked gene list $S=\left\{g_{1^{\prime }},g_{2^{\prime }},\ldots ,g_{r^{\prime }},\ldots ,g_{N^{\prime }}\right\}\;$ was obtained. The top ranked genes had strong relevance to OA but little redundancy among each other. In the next step, we further optimized the top 300 mRMR genes and got the final OA biomarker.

### Osteoarthritis biomarker optimization

Although the mRMR method can rank genes effectively, it is still unknown how many genes should be finally selected as the OA biomarker. Therefore, we applied a greedy method called incremental feature selection (IFS) (Jiang et al., 2013; Li et al., 2014; Shu et al., 2014; Zhang N. et al., 2014a; Huang et al., 2015; Zhang et al., 2015; Chen et al., 2018a) to optimize the number of signature genes. In this method, too few genes may miss the important information and too many genes may introduce noise.

During the IFS procedure, different numbers of genes were tried and their performances were evaluated. As there were too many combinations and the mRMR have already ranked the genes meaningfully, the mRMR genes were tested sequentially, i.e., in the r rounds, $\left\{g_{1^{\prime }},g_{2^{\prime }},\ldots ,g_{r^{\prime }}\right\}$ were tested. For each round, an SVM classifier was constructed based on the selected genes and its performance was evaluated through LOOCV. We used the R function SVM from package e1017 with default parameters and kernel of radial to build the SVM classifier.

To have a complete measurement of the prediction performance, four statistics, which were the sensitivity (Sn), specificity (Sp), accuracy (ACC), and Matthew's correlation coefficient (MCC), were calculated:

(4) $$\begin{array}{rcl} S_{n} & = & \frac{TP}{TP+FN} \end{array}$$

(5) $$\begin{array}{rcl} S_{p} & = & \frac{TN}{TN+FP} \end{array}$$

(6) $$\begin{array}{rcl} ACC & = & \frac{TP+TN}{TP+TN+FP+FN} \end{array}$$

(7) $$\begin{array}{rcl} MCC & = & \frac{TP\times TN-FP\times FN}{\sqrt{\left(TP+FP\right)\left(TP+FN\right)\left(TN+FP\right)\left(TN+FN\right)}} \end{array}$$

In Equations (4–7), TP, TN, FP, and FN were the number of true OA, true control, false OA, and false control samples, respectively.

On the basis of IFS results, we can determine how many genes should be chosen finally as the OA biomarker to achieve the best performance. As the numbers of OA samples and control samples were not balanced, the MCC was used as the main measurement for classification performance.

## Results

### The osteoarthritis-associated genes selected and ranked based on the mRMR method

To identify the OA-associated genes, we used the mRMR method that can select and rank genes based on their relevance with OA and their redundancy with other genes. The top 300 most discriminative genes for OA were selected and ranked using the mRMR method. These 300 mRMR genes will be further optimized using the IFS method.

### The osteoarthritis biomarker optimization based on the IFS method

**Figure 1:**
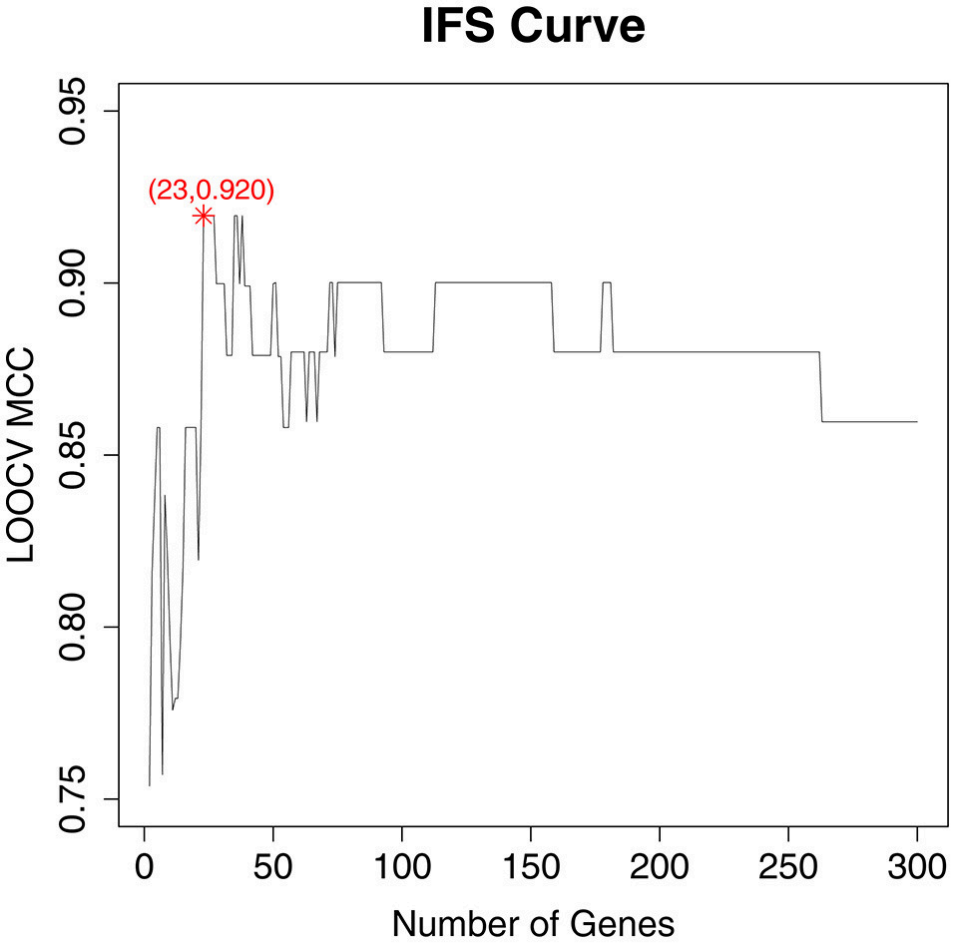
The IFS curve with the number of genes and the performance of classifiers. The *x*-axis was the number of genes used for SVM classifier construction and the *y*-axis was the classification Mathew's correlation coefficient (MCC) of the SVM classifier evaluated with Leave-One Out-Cross Validation (LOOCV). The peak of the IFS curve was MCC of 0.920 when 23 genes were used. The sensitivity, specificity, and accuracy of the 23-gene classifier were 0.991, 0.909, and 0.971, respectively.

As a ranked gene list, the top 300 mRMR genes included the candidate OA biomarker genes. However, we still did not know how many genes should be finally selected. To optimize OA biomarker selection, we tried different number of top genes and calculated their prediction performance. On the basis of these performances, we plotted an IFS curve, as shown in Figure 1, in which the *x*-axis was the number of genes and the *y*-axis was the LOOCV MCC of the SVM classifier. It can be seen that when the top 23 mRMR genes were used, the MCC was the highest, i.e., 0.920. Meanwhile, the sensitivity, specificity, and accuracy of the 23-gene classifier were 0.991, 0.909, and 0.971, respectively. The 23 genes are listed in Table 1. The confusion matrix of the predicted and actual sample classes is given in Table 2.

**Table 1:** The 23 osteoarthritis biomarker genes.

**Rank**	**Name**	**mRMR score**
1	SERINC3	0.298
2	ADRB2	0.153
3	NUFIP2	0.100
4	UBXD8	0.08
5	MLLT6	0.081
6	TNFSF14	0.083
7	APP	0.088
8	H3F3B	0.095
9	MFAP1	0.085
10	TAOK1	0.088
11	MTSS1	0.075
12	UPF1	0.084
13	C17orf91	0.079
14	GNL3L	0.072
15	ZNF20	0.074
16	RNF34	0.075
17	SNORD38A	0.072
18	PVRIG	0.073
19	CEP250	0.077
20	LRRC33	0.073
21	COG5	0.075
22	CDC2L5	0.071
23	PELO	0.073

**Table 2:** The confusion matrix of the predicted and actual sample classes.

	**Actual OA**	**Actual control**
Predicted OA	105	3
Predicted control	1	30
Sensitivity: 0.991	Specificity: 0.909	Accuracy: 0.971

**Figure 2:**
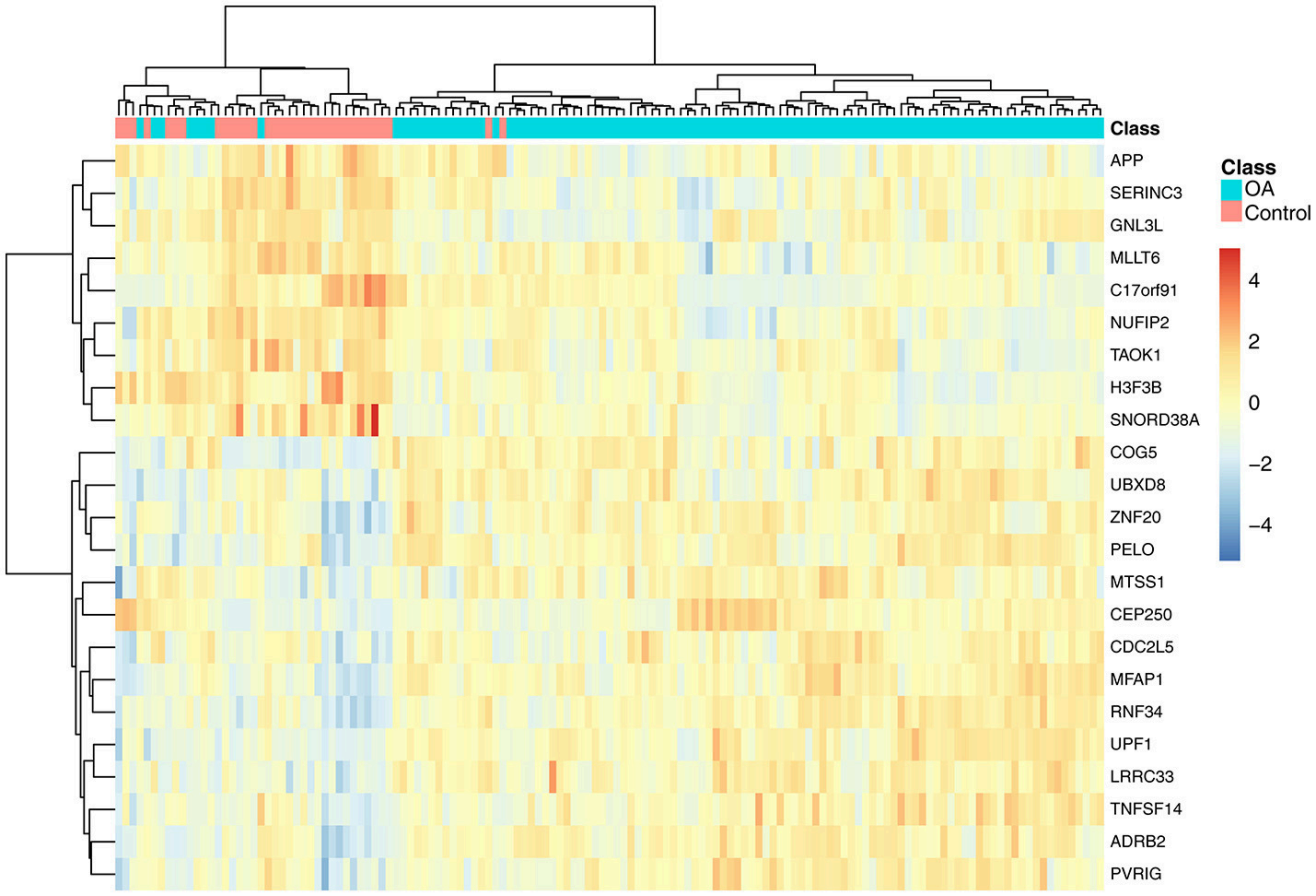
The heatmap of the 23 genes in osteoarthritis and control samples. Each row represented the expression level of one gene. The warm colors meant high expression and the cold colors meant low expression. The red and green columns were osteoarthritis and healthy samples, respectively. It can be seen that the osteoarthritis and control samples had very different expression patterns.

To investigate the associations of the 23 genes with OA, we plotted the heatmap of the 23 genes in OA and control samples, as shown in Figure 2. It can be seen that the OA and control samples had very different expression patterns. Generally speaking, APP, SERINC3, GNL3L, MLLT6, C17orf91, NUFIP2, TAOK1, H3F3B, and SNORD38A were highly expressed in control samples, whereas COG5, UBXD8, ZNF20, PELO, MTSS1, CEP250, CDC2L5, MFAP1, RNF34, UPF1, LRRC33, TNFSF14, ADRB2, and PVRIG were highly expressed in OA samples.

**Figure 3:**
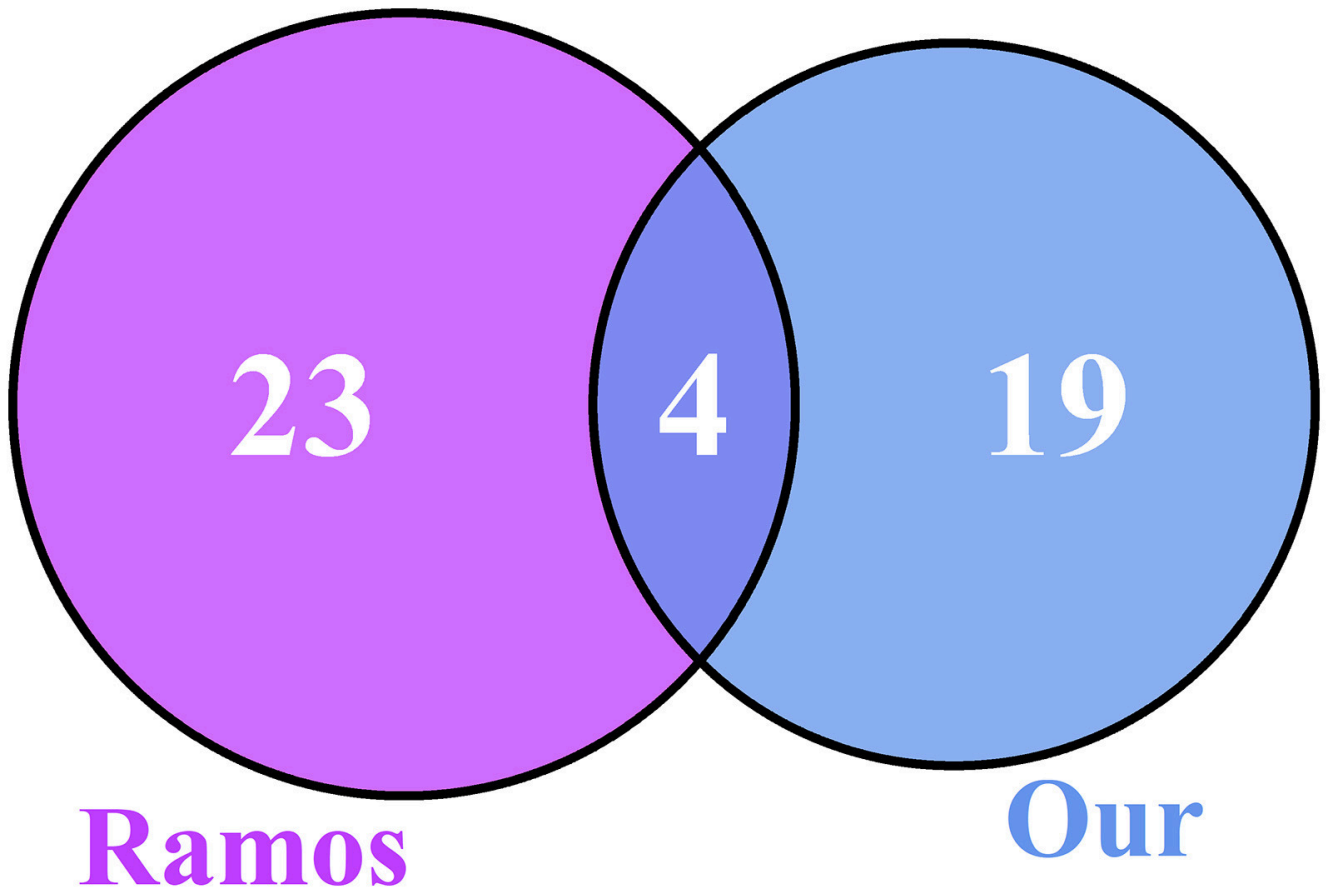
The Venn diagram of our 23 genes and the 27 genes from Ramos et al. (2014). There were four overlapped genes, ADRB2, H3F3B, PELO, and ZNF20, between the 23 osteoarthritis biomarker genes we identified and the 27 genes from Ramos et al. (2014). To evaluate the significance of overlap, we calculated the hypergeometric test *p-*value and odds ratio, which were 9.18e-09 and 229.87, respectively. The overlap was very significant.

We compared our 23 genes with the 27 genes from Ramos et al. (2014) and plotted the Venn diagram, as shown in Figure 3. There were four overlapped genes: ADRB2, H3F3B, PELO, and ZNF20. We evaluated the significance of overlapping using the hypergeometric test. The *p*-value was 9.18e-09 and the odds ratio was 229.87. The overlap between our 23 genes and the 27 genes from Ramos et al. (2014) was very significant.

### The functional analysis of the optimal osteoarthritis biomarker

**Figure 4:**
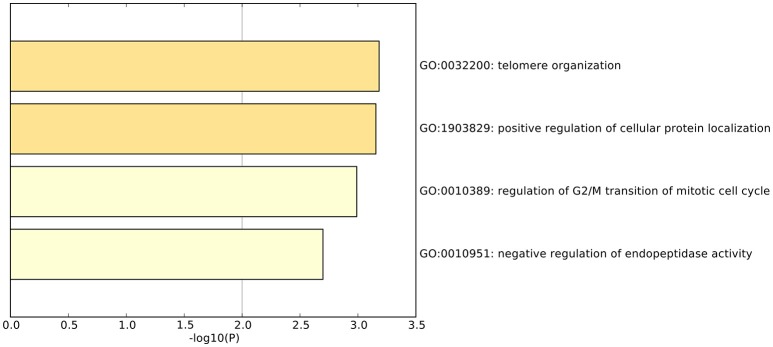
The enriched GO terms of the 23 osteoarthritis biomarker genes. The 23 osteoarthritis biomarker genes were enriched onto GO terms, such as GO:0032200: telomere organization, GO:1903829: positive regulation of cellular protein localization, GO:0010389: regulation of G2/M transition of mitotic cell cycle, and GO:0010951: negative regulation of endopeptidase activity.

We did functional enrichment analysis of 23 OA biomarker genes using Metascape (Tripathi et al., 2015). The Gene Ontology (GO) results are shown in Figure 4. The enriched GO terms were GO:0032200: telomere organization, GO:1903829: positive regulation of cellular protein localization, GO:0010389: regulation of G2/M transition of mitotic cell cycle, and GO:0010951: negative regulation of endopeptidase activity.

There have been many studies about the relationship between telomere length and OA (Kuszel et al., 2015; Wiwanitkit, 2017). OA is a typical geriatric disease and the telomere length becomes shorter and shorter during aging. In patients with OA, the shortening of telomeres was accelerated (Kuszel et al., 2015). H3F3B, UPF1, and GNL3L were involved in GO:0032200: telomere organization.

The dysfunctional regulation of cellular protein localization in OA was reasonable. Osteoarthritis is a joint disease and the gap junctional communication is regulated by the extracellular signal pathway (Niger et al., 2009). APP, TNFSF14, CEP250, and GNL3L were involved in GO:1903829: positive regulation of cellular protein localization.

There have been many theories about cell cycle and OA. Franke et al. found that during the pathogenesis of OA, advanced glycation end products (AGEs) influence osteoarthritic fibroblast-like synovial cells through inducing cell cycle arrest (Niger et al., 2009). de Andrés et al. discovered that the demethylation of an NF-κB enhancer can induce OA by regulating the cell cycle (de Andrés et al., 2016). APP, CEP250, and TAOK1 were involved in GO:0010389: regulation of the G2/M transition of the mitotic cell cycle.

It is known that several endogenous peptides have strong inflammatory effects in the joint and they are regulated by endopeptidase (Solan et al., 1998). Therefore, the genes from GO:0010951: negative regulation of endopeptidase activity, such as APP, TNFSF14, and RNF34, may play regulatory roles in OA.

### The protein interactions between the optimal osteoarthritis biomarkers

**Figure 5:**
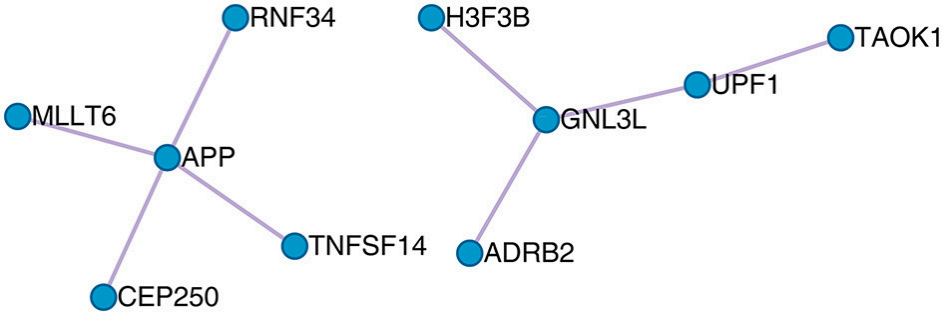
The PPI network of the 23 osteoarthritis biomarker genes. The 23 osteoarthritis biomarker genes formed two PPI clusters: the APP cluster that included APP, RNF34, TNFSF14, CEP250, and MLLT6, and the GNL3L cluster that included GNL3L, UPF1, TAOK1, ADRB2, and H3F3B.

The protein–protein interaction (PPI) between the optimal OA biomarker was derived from the STRING database (https://string-db.org/) and is shown in Figure 5. STRING is a comprehensive database that integrates protein functional associations from multiple sources, such as experiment and literature (Szklarczyk et al., 2015). From Figure 5, we can see that APP, RNF34, TNFSF14, CEP250, and MLLT6 formed a cluster and GNL3L, UPF1, TAOK1, ADRB2, and H3F3B formed another cluster.

Basically, the functions of the APP cluster that included APP, RNF34, TNFSF14, CEP250, and MLLT6 were regulation of endopeptidase activity, cell cycle, and cellular protein localization, whereas the functions the GNL3L cluster that included GNL3L, UPF1, TAOK1, ADRB2, and H3F3B were involved in telomere organization and cellular protein localization. Common function that linked the two clusters was cellular protein localization, which indicated that the secretion of protein into extracellular synovia was the key processes of OA.

## Discussion

As a common geriatric disease, OA has extremely high incidence, especially in elder people. As the chances of full recovery from late-stage OA are minimal, the most effective way of fighting OA is early diagnosis and early intervention. As a popular noninvasive test, liquid biopsy showed great potential in cancer detection. To identify the blood gene expression signature for OA, we studied the blood gene expression profiles of 106 patients with OA and 33 control samples. With mRMR and IFS methods, we identified 23 genes whose sensitivity, specificity, accuracy, and Mathew's correlation coefficient were 0.991, 0.909, 0.971, and 0.920, respectively. The prediction performance was excellent. The biological function analysis of these 23 genes suggested that there were two pathways or PPI modules associated with OA through aging, cellular protein localization, and inflammation. These findings may be helpful for understanding OA.

There were still some disadvantages of this work. Here, we investigated only the gene expression levels. However, recent studies have suggested that the genome-wide association study (GWAS) and epigenetics approaches were also effective in OA mechanisms (Kerkhof et al., 2010; Panoutsopoulou et al., 2011; Rushton et al., 2014; Ramos and Meulenbelt, 2017; Simon and Jeffries, 2017). Integrating the genetic and epigenetic data with gene expression may provide a more comprehensive view of OA. We surveyed the identified genes based on one expression and found that the variant rs3815148 of COG5 was found to be associated with OA by GWAS reports (Kerkhof et al., 2010; Panoutsopoulou et al., 2011). Rushton et al. reported that the methylation status of MLLT6, TNFSF14, TAOK1, and MTSS1 was different between OA hip subtypes and LRRC33 was hypermethylated in OA hip than OA knee (Rushton et al., 2014). These results encourage us and others to do integrative studies of multiomics data in OA in future.

## Data availability statement

The datasets for this study can be found in the Gene Expression Omnibus [https://www.ncbi.nlm.nih.gov/geo/query/acc.cgi?acc=GSE48556].

## Author contributions

JL and TH conceived and designed the experiments; JL performed the experiments; JL, C-NL, YK, and S-SF analyzed the data; JL and TH wrote the paper.

### Conflict of interest statement

The authors declare that the research was conducted in the absence of any commercial or financial relationships that could be construed as a potential conflict of interest.

## References

[B1] Aaron R. K. Racine J. R. Voisinet A. Evangelista P. Dyke J. P. (2018). Subchondral bone circulation in osteoarthritis of the human knee. Osteoarthritis Cartilage 26, 940–944. 10.1016/j.joca.2018.04.003 29723635

[B2] Ahmed U. Anwar A. Savage R. S. Thornalley P. J. Rabbani N. (2016). Protein oxidation, nitration and glycation biomarkers for early-stage diagnosis of osteoarthritis of the knee and typing and progression of arthritic disease. Arthritis Res. Ther. 18, 250. 10.1186/s13075-016-1154-3 27788684 PMC5081671

[B3] Appleton C. T. (2017). Osteoarthritis year in review 2017: biology. Osteoarthritis Cartilage 26, 296–303. 10.1016/j.joca.2017.02.024 29061493

[B4] Bay-Jensen A. C. Thudium C. S. Mobasheri A. (2018). Development and use of biochemical markers in osteoarthritis: current update. Curr. Opin. Rheumatol. 30, 121–128. 10.1097/BOR.0000000000000467 29040157

[B5] Budd E. Nalesso G. Mobasheri A. (2017). Extracellular genomic biomarkers of osteoarthritis. Expert. Rev. Mol. Diagn. 18, 55–74. 10.1080/14737159.2018.1415757 29235389

[B6] Cai Y. D. Huang T. Feng K. Y. Hu L. Xie L. (2010). A unified 35-gene signature for both subtype classification and survival prediction in diffuse large B-Cell lymphomas. PLoS ONE 5:12726. 10.1371/journal.pone.0012726 20856936 PMC2938343

[B7] Chen L. Li J. Zhang Y. H. Feng K. Wang S. Zhang Y.. (2018a). Identification of gene expression signatures across different types of neural stem cells with the Monte-Carlo feature selection method. J. Cell. Biochem 119, 3394–3403. 10.1002/jcb.26507 29130544

[B8] Chen L. Pan X. Hu X. Zhang Y. H. Wang S. Huang T.. (2018b). Gene expression differences among different MSI statuses in colorectal cancer. Int. J. Cancer 10.1002/ijc.31554. [Epub ahead of print]. 29696646

[B9] Costa-Cavalcanti R. G. da Cunha de Sá-Caputo D. Moreira-Marconi E. Ribeiro Kütter C. Brandão-Sobrinho-Neto S. Liane Paineiras-Domingos L.. (2018). Effect of auriculotherapy on the plasma concentration of biomarkers in individuals with knee osteoarthritis. J. Acupunct. Meridian Stud. 10.1016/j.jams.2018.05.005. [Epub ahead of print]. 29783048

[B10] de Andrés M. C. Takahashi A. Oreffo R. O. (2016). Demethylation of an NF-kappaB enhancer element orchestrates iNOS induction in osteoarthritis and is associated with altered chondrocyte cell cycle. Osteoarthritis Cartilage 24, 1951–1960. 10.1016/j.joca.2016.06.002 27307355

[B11] Feng J. Xia Y. Yuan L. Chen A. Yang N. Xiang Y. (2015). [An increased level of interleukin 27 in peripheral blood mononuclear cells and fibroblasts like synoviocytes of patients with rheumatoid arthritis or osteoarthritis]. Xi Bao Yu Fen Zi Mian Yi Xue Za Zhi 31, 1673–1676. 26648303

[B12] Fotouhi A. Maleki A. Dolati S. Aghebati-Maleki A. Aghebati-Maleki L. (2018). Platelet rich plasma, stromal vascular fraction and autologous conditioned serum in treatment of knee osteoarthritis. Biomed. Pharmacother. 104, 652–660. 10.1016/j.biopha.2018.05.019 29803179

[B13] Huang T. Cai Y. D. (2013). An information-theoretic machine learning approach to expression QTL analysis. PLoS ONE 8:e67899. 10.1371/journal.pone.0067899 23825689 PMC3692482

[B14] Huang T. Shu Y. Cai Y. D. (2015). Genetic differences among ethnic groups. BMC Genomics 16:1093. 10.1186/s12864-015-2328-0 26690364 PMC4687076

[B15] Huang T. Tu K. Shyr Y. Wei C. C. Xie L. Li Y. X. (2008). The prediction of interferon treatment effects based on time series microarray gene expression profiles. J. Transl. Med. 6:44. 10.1186/1479-5876-6-44 18691426 PMC2546378

[B16] Jiang Y. Huang T. Chen L. Gao Y. F. Cai Y. Chou K. C. (2013). Signal propagation in protein interaction network during colorectal cancer progression. Biomed. Res. Int. 2013:287019. 10.1155/2013/287019 23586028 PMC3615629

[B17] Kerkhof H. J. Lories R. J. Meulenbelt I. Jonsdottir I. Valdes A. M. Arp P.. (2010). A genome-wide association study identifies an osteoarthritis susceptibility locus on chromosome 7q22. Arthritis Rheum. 62, 499–510. 10.1002/art.27184 20112360 PMC3354739

[B18] Kraus V. B. Collins J. E. Hargrove D. Losina E. Nevitt M. Katz J. N.. (2017). Predictive validity of biochemical biomarkers in knee osteoarthritis: data from the FNIH OA Biomarkers Consortium. Ann. Rheum. Dis. 76, 186–195. 10.1136/annrheumdis-2016-209252 27296323 PMC5851287

[B19] Kuszel L. Trzeciak T. Richter M. Czarny-Ratajczak M. (2015). Osteoarthritis and telomere shortening. J. Appl. Genet. 56, 169–176. 10.1007/s13353-014-0251-8 25366419 PMC4412548

[B20] Li B. Q. You J. Huang T. Cai Y. D. (2014). Classification of non-small cell lung cancer based on copy number alterations. PLoS ONE 9:e88300. 10.1371/journal.pone.0088300 24505469 PMC3914971

[B21] Li F. Li C. Wang M. Webb G. I. Zhang Y. Whisstock J. C.. (2015). GlycoMine: a machine learning-based approach for predicting N-, C- and O-linked glycosylation in the human proteome. Bioinformatics 31, 1411–1419. 10.1093/bioinformatics/btu852 25568279

[B22] Li J. Huang T. (2017). Predicting and analyzing early wake-up associated gene expressions by integrating GWAS and eQTL studies. Biochim. Biophys. Acta 1864(6 Pt B), 2241–2246. 10.1016/j.bbadis.2017.10.036 29109033

[B23] Liu L. Chen L. Zhang Y. H. Wei L. Cheng S. Kong X.. (2017). Analysis and prediction of drug-drug interaction by minimum redundancy maximum relevance and incremental feature selection. J. Biomol. Struct. Dyn. 35, 312–329. 10.1080/07391102.2016.1138142 26750516

[B24] Lorenzo P. Aspberg A. Saxne T. Önnerfjord P. (2017). Quantification of cartilage oligomeric matrix protein (COMP) and a COMP neoepitope in synovial fluid of patients with different joint disorders by novel automated assays. Osteoarthritis Cartilage 25, 1436–1442. 10.1016/j.joca.2017.04.004 28473207

[B25] Martel-Pelletier J. Raynauld J. P. Dorais M. Abram F. Pelletier J. P. (2016). The levels of the adipokines adipsin and leptin are associated with knee osteoarthritis progression as assessed by MRI and incidence of total knee replacement in symptomatic osteoarthritis patients: a post hoc analysis. Rheumatology 55, 680–688. 10.1093/rheumatology/kev408 26660640

[B26] Nelson A. E. (2017). Osteoarthritis year in review 2017: clinical. Osteoarthritis Cartilage 26, 319–325. 10.1016/j.joca.2017.11.014 29229563 PMC5835411

[B27] Niger C. Howell F. D. Stains J. P. (2009). Interleukin-1beta increases gap junctional communication among synovial fibroblasts via the extracellular-signal-regulated kinase pathway. Biol. Cell 102, 37–49. 10.1042/BC20090056 19656083 PMC2874634

[B28] Niu B. Huang G. Zheng L. Wang X. Chen F. Zhang Y.. (2013). Prediction of substrate-enzyme-product interaction based on molecular descriptors and physicochemical properties. Biomed. Res. Int. 2013:674215. 10.1155/2013/674215 24455714 PMC3881445

[B29] Panoutsopoulou K. Southam L. Elliott K. S. Wrayner N. Zhai G. Beazley C.. (2011). Insights into the genetic architecture of osteoarthritis from stage 1 of the arcOGEN study. Ann. Rheum. Dis. 70, 864–867. 10.1136/ard.2010.141473 21177295 PMC3070286

[B30] Peng H. Long F. Ding C. (2005). Feature selection based on mutual information: criteria of max-dependency, max-relevance, and min-redundancy. IEEE Trans. Pattern Anal. Mach. Intell. 27, 1226–1238. 10.1109/TPAMI.2005.159 16119262

[B31] Qin W. Li Y. Li J. Yu L. Wu D. Jing R.. (2012). Predicting deleterious non-synonymous single nucleotide polymorphisms in signal peptides based on hybrid sequence attributes. Comput. Biol. Chem. 36, 31–35. 10.1016/j.compbiolchem.2011.12.001 22277674

[B32] Ramos Y. F. Bos S. D. Lakenberg N. Böhringer S. den Hollander W. J. Kloppenburg M.. (2014). Genes expressed in blood link osteoarthritis with apoptotic pathways. Ann. Rheum. Dis. 73, 1844–1853. 10.1136/annrheumdis-2013-203405 23864235

[B33] Ramos Y. F. Meulenbelt I. (2017). The role of epigenetics in osteoarthritis: current perspective. Curr. Opin. Rheumatol. 29, 119–129. 10.1097/BOR.0000000000000355 27749371

[B34] Rushton M. D. Reynard L. N. Barter M. J. Refaie R. Rankin K. S. Young D. A.. (2014). Characterization of the cartilage DNA methylome in knee and hip osteoarthritis. Arthritis Rheumatol. 66, 2450–2460. 10.1002/art.38713 24838673 PMC4314681

[B35] Schaefer L. F. Sury M. Yin M. Jamieson S. Donnell I. Smith S. E.. (2017). Quantitative measurement of medial femoral knee cartilage volume - analysis of the OA Biomarkers Consortium FNIH Study cohort. Osteoarthritis Cartilage 25, 1107–1113. 10.1016/j.joca.2017.01.010 28153788 PMC5466831

[B36] Shu Y. Zhang N. Kong X. Huang T. Cai Y. D. (2014). Predicting A-to-I RNA editing by feature selection and random forest. PLoS ONE 9:e110607. 10.1371/journal.pone.0110607 25338210 PMC4206426

[B37] Simon T. C. Jeffries M. A. (2017). The epigenomic landscape in osteoarthritis. Curr. Rheumatol. Rep. 19:30. 10.1007/s11926-017-0661-9 28456906 PMC5957081

[B38] Solan N. J. Ward P. E. Sanders S. P. Towns M. C. Bathon J. M. (1998). Soluble recombinant neutral endopeptidase (CD10) as a potential antiinflammatory agent. Inflammation 22, 107–121. 10.1023/A:1022304025789 9484654

[B39] Song J. Wang H. Wang J. Leier A. Marquez-Lago T. Yang B.. (2017). PhosphoPredict: a bioinformatics tool for prediction of human kinase-specific phosphorylation substrates and sites by integrating heterogeneous feature selection. Sci. Rep. 7:6862. 10.1038/s41598-017-07199-4 28761071 PMC5537252

[B40] Steinberg J. Ritchie G. R. S. Roumeliotis T. I. Jayasuriya R. L. Clark M. J. Brooks R. A.. (2017). Integrative epigenomics, transcriptomics and proteomics of patient chondrocytes reveal genes and pathways involved in osteoarthritis. Sci. Rep. 7:8935. 10.1038/s41598-017-09335-6 28827734 PMC5566454

[B41] Sun L. Yu Y. Huang T. An P. Yu D. Yu Z.. (2012). Associations between ionomic profile and metabolic abnormalities in human population. PLoS ONE 7:e38845. 10.1371/journal.pone.0038845 22719963 PMC3374762

[B42] Szklarczyk D. Franceschini A. Wyder S. Forslund K. Heller D. Huerta-Cepas J.. (2015). STRING v10: protein-protein interaction networks, integrated over the tree of life. Nucleic Acids Res. 43(Database issue), D447–D452. 10.1093/nar/gku1003 25352553 PMC4383874

[B43] Tripathi S. Pohl M. O. Zhou Y. Rodriguez-Frandsen A. Wang G. Stein D. A.. (2015). Meta- and orthogonal integration of influenza “OMICs” data defines a role for UBR4 in virus budding. Cell Host Microbe 18, 723–735. 10.1016/j.chom.2015.11.002 26651948 PMC4829074

[B44] Tusher V. G. Tibshirani R. Chu G. (2001). Significance analysis of microarrays applied to the ionizing radiation response. Proc. Natl. Acad. Sci. U.S.A. 98, 5116–5121. 10.1073/pnas.091062498 11309499 PMC33173

[B45] Vina E. R. Kwoh C. K. (2017). Epidemiology of osteoarthritis: literature update. Curr. Opin. Rheumatol. 30, 160–167. 10.1097/BOR.0000000000000479 29227353 PMC5832048

[B46] Wang D. Li J. R. Zhang Y. H. Chen L. Huang T. Cai Y. D. (2018). Identification of differentially expressed genes between original breast cancer and xenograft using machine learning algorithms. Genes 9:155. 10.3390/genes9030155 29534550 PMC5867876

[B47] Wang H. Feng L. Zhang Z. Webb G. I. Lin D. Song J. (2016). Crysalis: an integrated server for computational analysis and design of protein crystallization. Sci. Rep. 6:21383. 10.1038/srep21383 26906024 PMC4764925

[B48] Wasilko S. M. Tourville T. W. DeSarno M. J. Slauterbeck J. R. Johnson R. J. Struglics A.. (2016). Relationship between synovial fluid biomarkers of articular cartilage metabolism and the patient's perspective of outcome depends on the severity of articular cartilage damage following ACL trauma. J. Orthop. Res. 34, 820–827. 10.1002/jor.23084 26497486 PMC6533635

[B49] Wiwanitkit V. (2017). Telomere length and angiogenic cytokines in knee osteoarthritis. Int. J. Rheum. Dis. 20:2141. 10.1111/1756-185X.13140 28730662

[B50] Zhang N. Huang T. Cai Y. D. (2014a). Discriminating between deleterious and neutral non-frameshifting indels based on protein interaction networks and hybrid properties. Mol. Genet. Genomics 290, 343–352. 10.1007/s00438-014-0922-5 25248637

[B51] Zhang N. Wang M. Zhang P. Huang T. (2016). Classification of cancers based on copy number variation landscapes. Biochim. Biophys. Acta 1860(11 Part B), 2750–2755. 10.1016/j.bbagen.2016.06.003 27266344

[B52] Zhang N. Zhou Y. Huang T. Zhang Y. C. Li B. Q. Chen L.. (2014b). Discriminating between lysine sumoylation and lysine acetylation using mRMR feature selection and analysis. PLoS ONE 9:e107464. 10.1371/journal.pone.0107464 25222670 PMC4164654

[B53] Zhang P. W. Chen L. Huang T. Zhang N. Kong X. Y. Cai Y. D. (2015). Classifying ten types of major cancers based on reverse phase protein array profiles. PLoS ONE 10:e0123147. 10.1371/journal.pone.0123147 25822500 PMC4378934

[B54] Zhang T. M. Huang T. Wang R. F. (2018). Cross talk of chromosome instability, CpG island methylator phenotype and mismatch repair in colorectal cancer. Oncol. Lett. 16, 1736–1746. 10.3892/ol.2018.8860 30008861 PMC6036478

[B55] Zhang X. Chen C. Wu M. Chen L. Zhang J. Zhang X.. (2012). Plasma microRNA profile as a predictor of early virological response to interferon treatment in chronic hepatitis B patients. Antivir. Ther. 17, 1243–1253. 10.3851/IMP2401 22997154

[B56] Zhang Y. Xu J. Zheng W. Zhang C. Qiu X. Chen K.. (2014). newDNA-Prot: prediction of DNA-binding proteins by employing support vector machine and a comprehensive sequence representation. Comput. Biol. Chem. 52, 51–59. 10.1016/j.compbiolchem.2014.09.002 25240115

[B57] Zhang Y. H. Huang T. Chen L. Xu Y. Hu Y. Hu L. D.. (2017). Identifying and analyzing different cancer subtypes using RNA-seq data of blood platelets. Oncotarget 8, 87494–87511. 10.18632/oncotarget.20903 29152097 PMC5675649

[B58] Zhao T. H. Jiang M. Huang T. Li B. Q. Zhang N. Li H. P.. (2013). A novel method of predicting protein disordered regions based on sequence features. Biomed Res. Int. 2013:414327. 10.1155/2013/414327 23710446 PMC3654632

[B59] Zhou Y. Zhang N. Li B. Q. Huang T. Cai Y. D. Kong X. Y. (2015). A method to distinguish between lysine acetylation and lysine ubiquitination with feature selection and analysis. J. Biomol. Struct. Dyn. 33, 2479–2490. 10.1080/07391102.2014.1001793 25616595

